# Numerical simulation of strength and failure analysis of heterogeneous sandstone under different loading rates

**DOI:** 10.1038/s41598-023-50048-w

**Published:** 2023-12-20

**Authors:** Weihao Zhu, Feng Wang, Jun Mu, Dawei Yin, Lang Lu, Zetao Chen

**Affiliations:** 1 College of Energy and Mining Engineering, Shandong University of Science and Technology, Qingdao, 266590 Shandong China; 2 State Key Laboratory of Mining Disaster Prevention and Control Co-founded by Shandong Province and the Ministry of Science and Technology, Shandong University of Science and Technology, Qingdao, 266590 Shandong China; 3 Jinneng Holding Lu’an Xinjiang Coal Chemical (Group) Co., Ltd, Hami, Xinjiang, 839000 China

**Keywords:** Engineering, Civil engineering

## Abstract

Natural rock masses often contain heterogeneous structures with varying sizes, non-uniform distributions, and strengths, which influence the mechanical response characteristics and crack propagation modes under loading. Furthermore, heterogeneous structures can affect the stability of the rock mass, in serious cases, leading to geotechnical and mining engineering disasters. In the present work, a parallel-bond model (PBM)-based numerical simulation using Particle Flow Code (PFC) was carried out to study the strength and failure characteristics of sandstone specimens with heterogeneous structures under different loading rates. The results show that the peak strength increases with the increasing loading rate. In addition, all of the initial cracks occurred at the edges of the heterogeneous structures of specimens under different loading rates. The greater the loading rate, the greater the stress concentration degree at the edge of the heterogeneous structures, the greater the dissipated energy as the sandstone specimens with heterogeneous structures suffer damage, the more intense the acoustic emission activity, and the greater the damage degree of the specimens. The number of cracks generated in sandstone specimens with heterogeneous structures increases gradually with the increasing loading rate during the initial loading stage, and gradually decreases after the specimens are damaged. Cracks propagate and develop from the upper right region to the lower right region of the specimens, forming crack groups that rapidly penetrate the specimens, leading to failure. Under different loading rates, the final failure behavior of the sandstone specimens with heterogeneous structures changes from an inverted V-type to θ-type, then gradually evolves to O-type failure.

## Introduction

As natural geological materials, rocks exhibit obvious heterogeneity in both physical and mechanical properties due to differences in particle sizes, particle shapes, and the type and distribution of rock-forming minerals. The deformation and strength characteristics of rocks are largely controlled by internal heterogeneous structures of the rock^1–5^. Under external forces, the heterogeneous structures lead to an uneven distribution of the stress field inside the rock, which will affect the mechanical properties and mechanical behavior of rock under loading, thereby influencing the fracture and failure evolution process of the rock^6–8^. In addition, changes in the rock loading rate induced by construction, loading, structural compression, etc. will also have an important influence on the fracture and failure evolution processes of rock^9–11^.

Extensive experimental studies have been performed to investigate the mechanical behavior of rock under different loading rates including the strength characteristics^12–16^, deformation and damage characteristics^17–21^, energy evolution^22–24^, crack development and propagation^25^, and other characteristics^26,27^ during the failure process. However, due to the discrete distribution of heterogeneous structures in the rock, the experiment cannot accurately located, which makes rock mass exhibit more complex failure modes and anisotropic strength characteristics, the development and propagation of cracks in rock are difficult to observe. Numerical simulations based on particle flow theory can effectively solve this problem, and have become one of the most widely used methods for studying crack initiation and propagation in the failure processes of brittle materials such as rocks^28^. For example^29^, simulated the uniaxial compression test of coal-rock composite layer by PFC2D software, focusing on the laws of mechanical behavior of composite layer under different loading rates. Tang et al.^30^ studied the laws of the shear stiffness, the peak shear strength and displacement of artificial rocks under different loading rates through direct shear experiments. Zhang et al.^31^ conducted Brazilian splitting and uniaxial compression tests on granite under different loading rates and quantitatively analyzed the effect of loading rates on stress–strain, fracture morphology, strain energy, and acoustic emission behaviors using particle flow theory. Huang et al.^32^ proposed a method of preparing coal samples with initial damage by cyclic pre-loading and unloading as to investigate the mechanical and creep properties of the initially damaged coal specimen. Zhou et al.^33^ carried out the numerical test under true triaxial dynamic cyclic loading of a hard rock at different loading rates by a dynamic constitutive model for rock materials. Huang et al.^34^ carried out simulations of the loading of rock-like specimens with two non-parallel cracks, and analyzed the stress–strain curves, mechanical properties, acoustic emission events, crack behavior, and energy characteristics of test specimens with preset cracks under different loading rates. The macroscopic mechanical behavior and microscopic damage mechanism of the test specimens were revealed.

The above studies mainly focused on the mechanical properties and failure evolution laws of conventional rocks under different loading rates. However, natural rock masses often contain heterogeneous structures such as pyrite in coal, quartz in granite, and gypsum in dolomite. Although these heterogeneous structures can affect the mechanical response characteristics and crack propagation mode of the rock mass, few reports on these phenomena can be found in the literature. In this paper, the mechanical and failure behavior of sandstone specimens with heterogeneous structures under different loading rates were studied through numerical simulations based on the parallel-bond model (PBM) using PFC. The effects of loading rate on the stress–strain, dissipated energy, acoustic emission event count, and failure characteristics of sandstone specimens with heterogeneous structures were analyzed.

## Numerical models

### Generation of particles

**Figure 1:**
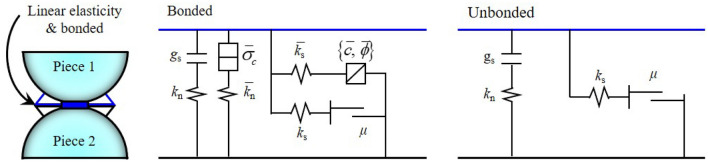
The parallel-bond model (PBM).

Particle Flow Code is a simulation software set that can be used to investigate the macroscopic mechanical behavior of numerical models of discontinuous media through changes in the mesoscopic parameters of particles based on particle discrete element theory^35^. The PFC software includes two types of bonding models: the contact-bond model (CBM) and the PBM. The PBM is more suitable for simulating the characteristics of rock-like materials because both force and torque can be transmitted and together, the contact stiffness and bonding stiffness constitute the macroscopic stiffness. The PBM constitutive model is shown in Fig. 1. When particles are bonded, they can resist torque and will exhibit linear elasticity until their strength limit is exceeded, and the bonding model suffers failure.

To further study the effects of loading rate on the failure mechanism of sandstone specimens with heterogeneous structures, a two-dimensional PBM was established using the PFC particle discrete-element modelling software. The built-in FISH programming language was used. Furthermore, using the PBM, the random distribution of heterogeneous structures in sandstone was considering and interrelated particles were approximately bonded into flexible clusters to simulate meso-characteristics with clustering. The specific formation of the sandstone specimens with heterogeneous structures using PFC can be described as follows.

**Figure 2:**
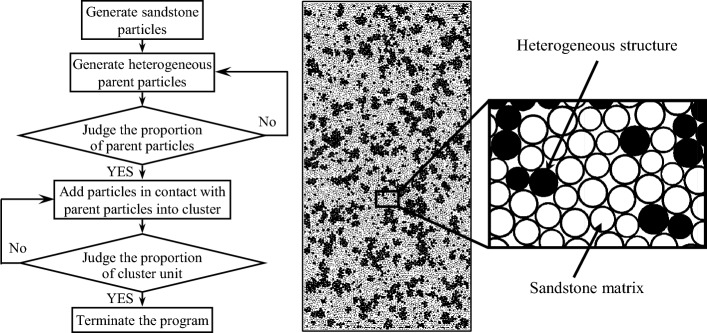
Numerical model and its generating process.

1. A total of 11,603 particles were formed in a 50 mm × 100 mm area by performing an initial compression, exerting an initial isotropic stress, deleting suspended particles, assigning values to parallel-bond parameters, and some additional steps. Through calculations, a good contact model between the generated particles was formed and random clusters were generated. Supposing all particles are sandstone particles, the current total area of particles can be calculated and is defined by two variables: heterogeneous particle area and heterogeneous particle number. In this case, the values of the two variables were zero and heterogeneous particle generation was controlled using the ratio of the heterogeneous particle areas.
2. To ensure the distribution of heterogeneous structures was random, randomly selected particles in the specimen area were first defined as heterogeneous structures parent particles. Then, a uniform distribution function in FISH was used to determine whether the number of generated heterogeneous parent particles and their area ratios met the requirements of a random distribution, in other words, whether the content of the parent particles reached 10% of the total particles. Parent particles were continuously generated until these requirements were satisfied. In this way, unaggregated parent particles with a specific area ratio were generated.
3. All particles in contact with parent particles were identified and added to the cluster unit in FISH, which was also used to assesses whether the particle number in the cluster unit has reached the set upper limit. The program continued to seek out particles in contact with the cluster unit until the upper limit was reached, and the particles were added to the cluster unit to achieve the clustering effect. The search ends when the upper limit is reached. During the heterogeneous particle clustering process, the area occupied by heterogeneous particles was constantly calculated and the upper limit of the area ratio of particles in a cluster unit indicated the heterogeneous particle content. When the number of particles in the specimen reached the expected content, the program ended and the generation of sandstone specimens with randomly distributed heterogeneous structures was complete. A flowchart of the process for generating sandstone specimens with heterogeneous structures and simulated specimens with 30% content are shown in Fig. 2.

### Parameters of models

**Figure 3:**
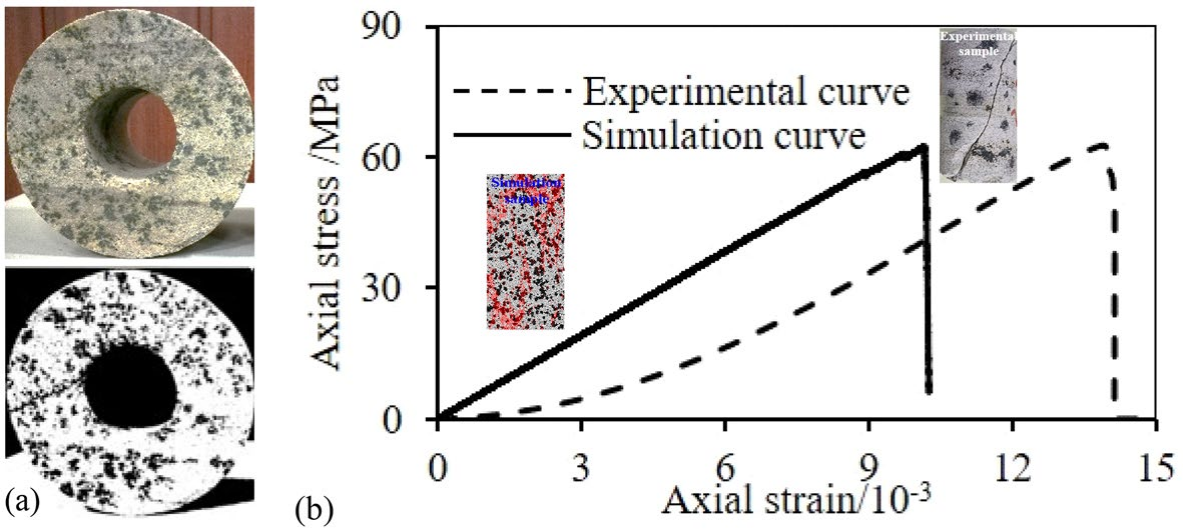
Parameters calibration process: (**a**) Binarization, (**b**) stress–strain curve and failure mode.

Since no direct quantitative relationship between the microscopic mechanical parameters can be established in numerical simulations with PFC, macroscopic mechanical parameters from laboratory tests were used to obtain reasonable meso-parameters. Before the numerical simulation, the results of uniaxial compression tests performed on complete specimens were used to repeatedly calibrate the meso-mechanical parameters in the numerical model under the same loading rate using the trial-and-error and control variable methods. To determine the to-be-calibrated heterogeneous structure content in specimens, the first step was to perform binarization processing on the three raw rocks used for specimens preparation, as shown in Fig. 3a, and the processed images were to create a computer-aided design (CAD) model, which was used to calculate the area ratio. The average content of heterogeneous structures was 29.3% based on the three rock specimens. For convenience, the area ratio of heterogeneous structures in the simulated specimens was set as 30%. The meso-parameters of the sandstone specimens with heterogeneous structures are shown in Table 1. The stress–strain curve and calibration results of the failure modes are shown in Fig. 3b. The peak strength, elastic modulus, and failure mode obtained through numerical simulation were consistent with the results of uniaxial compression tests. However, the stress–strain relationship curve obtained by numerical simulation is almost a straight line before the peak strength, and compared with the stress–strain relationship curve obtained by laboratory uniaxial compression test, it lacks the micro-crack compaction stage. This is because PFC, as a discrete element simulation software, simulates the motion and mechanical characteristics of discrete bodies by generating circular particles, which are rigid bodies, and there is no deformation of individual particles after being stressed, so there is no process of primary void compaction, so it cannot reflect the initial compaction of specimens in laboratory tests. Previous studies have demonstrated that the parallel-bond model in PFC can better reflect the bonding characteristics and macroscopic failure behavior of rock particles, and the parallel-bond model is adopted in most of the related types of studies^36–38^.The specific calibration process is as follows.

1. The elastic modulus was calibrated based on direct tensile conditions and the effective modulus of linear contact was assumed to be relatively small. By changing the effective parallel-bond modulus and taking higher values for the other parameters, the stress–strain curve under direct tension was obtained. Through fitting, the relationship between the tensile elastic modulus and the effective parallel-bond modulus was also obtained. Then the elastic modulus to be calibrated was substituted into the curve to obtain the parallel-bond modulus.
2. The linear contact modulus was calibrated using data from biaxial compression tests while keeping the parallel-bond modulus unchanged. The relationship between the linear contact modulus and the elastic modulus was obtained through fitting. Then, the linear contact modulus was obtained by substituting the elastic modulus to be calibrated.
3. Keeping the linear contact modulus and parallel-bond modulus unchanged and assuming that the stiffness ratios of the linear contact component and parallel-bond component are the same, the corresponding relationship between the stiffness ratio and macroscopic Poisson's ratio was obtained. Then, the stiffness ratio was obtained by substituting the Poisson's ratio to be calibrated.
4. The failure modes of specimens were calibrated by keeping all of the above-mentioned parameters unchanged and adjusting the bonding ratio. Finally, the parameters were adjusted slightly.

**Table 1:** Micro-Parameters of Specimens.

Parameters	Emod (GPa)	Krat	Fric	Pb_emod (GPa)	Pb_krat	Pb_ten (MPa)	Pb_coh (MPa)	pb_mcf
Rock matrix	1.4	1.5	0.5	3.0	1.5	16.0	25.0	0
Heterogeneous structure	0.7	1.5	0.5	1.5	1.5	8.0	12.5	0

### Numerical schemes

In this work, a numerical model of sandstone specimens with heterogeneous structures was established with PFC to study the influence of loading rate on the strength and deformation characteristics of sandstone with heterogeneous structures. To eliminate the influence of other factors on the simulation results, the particle size ratio, particle mechanical properties, and area ratio of heterogeneous structures to sandstone matrix were fixed in the model. Loading rates of 0.01 mm/s, 0.05 mm/s, 0.1 mm/s, 0.15 mm/s, 0.2 mm/s and 0.25 mm/s were considered. A program was written in FISH to monitor the stress–strain behavior, crack development and propagation process, acoustic emission, and energy changes caused by bond fracture of the specimens in real-time during the loading process.

## Results and discussion

### Strength of sandstone specimens with heterogeneous structures

**Figure 4:**
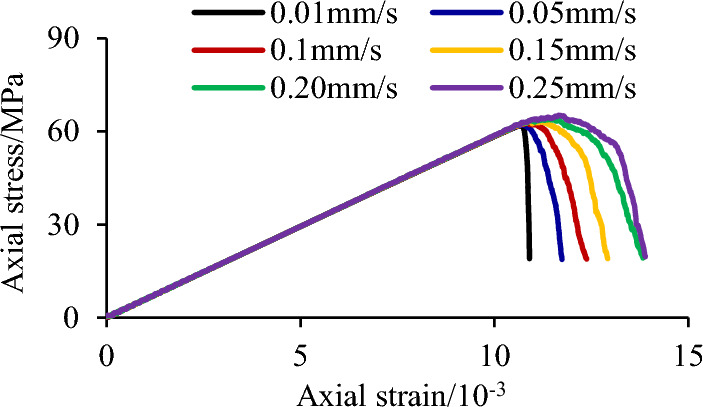
Peak strength curves of sandstone specimens with heterogeneous structures under different loading rates.

Figure 4 shows the variation of peak strength of sandstone specimens with heterogeneous structures under different loading rates. In the simulation process, we set two variables, instantaneous intensity and peak intensity, with the help of the FISH language. When the peak intensity is less than instantaneous intensity, the peak intensity is updated to instantaneous intensity, and when the peak intensity is greater than instantaneous intensity, the peak intensity remains unchanged, so as to obtain the peak intensity in real-time. After the specimen is damaged, judge whether the post-peak strength is less than 30% of the peak strength, and if so, stop loading. The average elastic modulus was 3.64 GPa. The peak strength gradually increased with the increasing loading rate.

### AE event count and dissipated energy of sandstone specimens with heterogeneous structures

**Figure 5:**
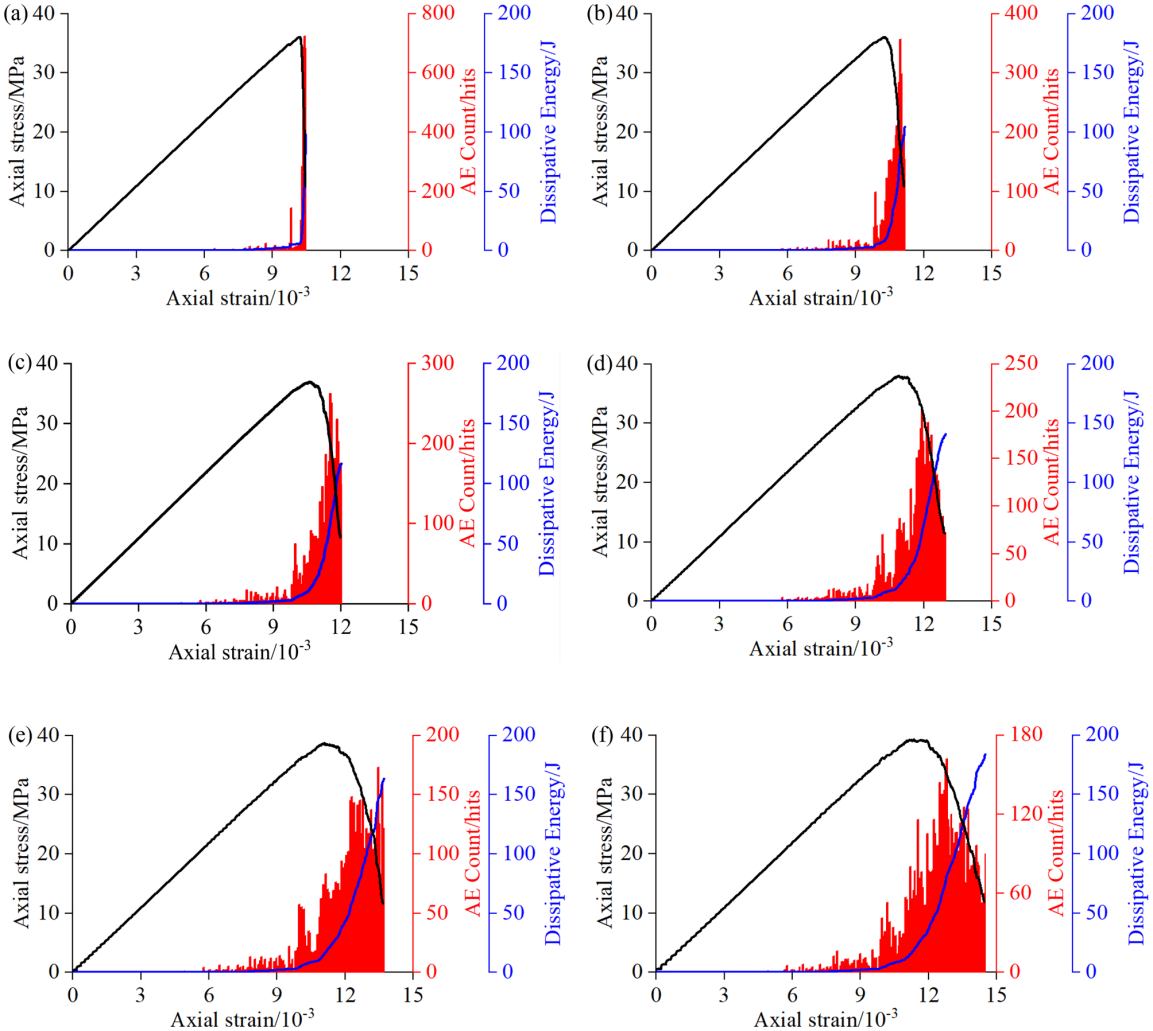
AE event count and dissipated energy curves of sandstone specimens with heterogeneous structures under different loading rates: (**a**) 0.01 mm/s, (**b**) 0.05 mm/s, (**c**) 0.10 mm/s, (**d**) 0.15 mm/s, (**e**) 0.20 mm/s, (**f**) 0.25 mm/s.

The acoustic emission phenomenon in the loading process, dissipated energy in the deformation and failure processes of sandstone specimens with heterogeneous structures were monitored in real-time using a built-in FISH program in PFC. In this paper, the specific process of acoustic emission simulation is as follows. Firstly, the deformation process of the specimen during loading is calculated by the FISH language. On this basis, taking the strain of the specimen as a reference, the acoustic emission collection interval is set to a minimum strain value, and the variation of crack number before and after sampling is calculated as the number of acoustic emission events during loading. Figure 5 shows the number of acoustic emission events and variation of dissipated energy of the sandstone specimens with heterogeneous structures under different loading rates.

1. During the elastic deformation stage, when the axial stress of sandstone specimens with heterogeneous structures is less than the crack initiation stress, the specimen is in the elastic compression state, no cracks are generated inside the specimen, and the number of acoustic emission events and dissipated energy are both zero. As loading continues, the axial stress will gradually exceed the crack initiation stress and the strength of the heterogeneous structures in the specimen will be lower than that of the sandstone matrix. In this case, few cracks are generated in the specimen, which develop and propagate only in heterogeneous structures, the number of acoustic emission events generally increases, the sudden increase points rose, and the dissipated energy also gradually increases, however, the growth rate is relatively low.
2. During the unstable crack propagation stage, the axial stress gradually exceeds the damage stress. Cracks in the sandstone specimen with heterogeneous structures increase rapidly and gradually propagate into the sandstone matrix. When the loading rate was 0.01 mm/s and 0.05 mm/s, cracks inside the heterogeneous structures had sufficient time to evolve and propagate and the internal structures were readjusted in time to accommodate the axial stress. The stress decreased significantly before reaching the peak strength, and the number of acoustic emission events increased sharply and the dissipated energy rose in a stepwise manner. When the loading rate was 0.1 mm/s, 0.15 mm/s, 0.2 mm/s, and 0.25 mm/s, the specimens could not sufficiently adapt and the stress–strain curve was relatively smooth before the peak strength.
3. During the post-peak failure stage, the axial stress gradually exceeds the peak strength, a large number of cracks are generated inside the specimen and rapidly penetrate the sandstone matrix until the specimen finally ruptures. When the loading rate was 0.01 mm/s and 0.05 mm/s, due to the rapid decline in stress, acoustic emission events were relatively sparsely distributed, the acoustic emission signals showed lower activity, and the dissipated energy increased rapidly. When the loading rate was 0.1 mm/s, 0.15 mm/s, 0.2 mm/s, and 0.25 mm/s, the rate of decrease in stress slowed, acoustic emission events were more densely distributed, the acoustic emission signal activity increased significantly, and the rate of increasing dissipated energy slowed down. The number of acoustic emission events reached a maximum near the peak of the stress–strain curve. With continuous loading, the number of acoustic emission events gradually decreased and remained stable at a lower level, while the dissipated energy continued to increase.

Therefore, at higher loading rates, the duration of loading is relatively short, which restricts the evolution and propagation of cracks inside heterogeneous structures. With the continuous accumulation of energy, a large amount of elastic energy is stored in the sandstone and released suddenly near the peak strength, resulting in more activity in the acoustic emission signal, and thus exhibiting more intense acoustic emission activities. Before the peak strength was reached, the dissipated energy increased more slowly as the loading rate increased. When the peak strength was reached, elastic energy stored in the sandstone was instantly released, resulting in a sharp increase in dissipated energy and instantaneous failure of the sandstone specimen. The larger the loading rate, the greater the dissipated energy, and the more energy is absorbed by cracks, allowing them to develop and propagate, leading to a greater failure degree.

### Crack growth and propagation of sandstone specimens with heterogeneous structures

**Figure 6:**
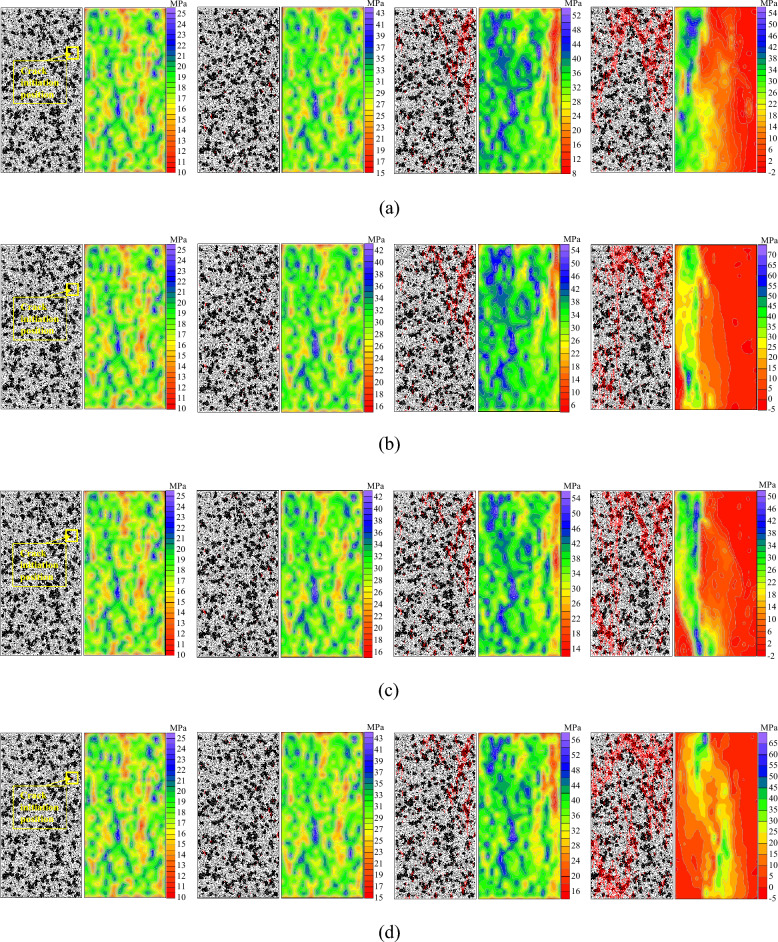

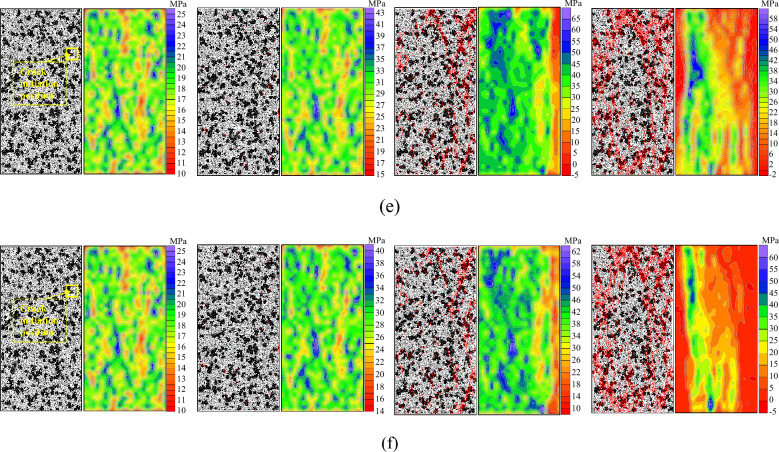
Crack growth and stress evolution of sandstone with heterogeneous structures under different loading rates: (**a**) 0.01 mm/s, (**b**) 0.05 mm/s, (**c**) 0.10 mm/s, (**d**) 0.15 mm/s, (**e**) 0.20 mm/s, (**f**) 0.25 mm/s.

To study the law of crack development and propagation in sandstone specimens with the heterogeneous structures under different loading rates, firstly, we set the sampling interval by writing codes, take the crack increment of 10 as the sampling interval, sample every time the number of cracks increases by 10, and obtain a large number of pictures in the process of specimen simulation. Finally, taking the obvious crack expansion and stress distribution change as the standard, that is, the pictures of four characteristic moments, i.e., crack initiation, damage (the moment when the number of cracks in the model increases rapidly), peak value, and final failure, are selected for comparison to analyze the crack propagation law of the specimen, and the maximum principal stress of the sandstone specimen with heterogeneous structures is monitored in real-time based on the built-in FISH language of PFC, as shown in Fig. 6, which shows the crack development and stress evolution law of the sandstone specimen with the heterogeneous structures under different loading rates.

1. During the initial loading stage, the specimens are in the compaction state and the internal structure adjusts to the axial stress without obvious stress concentrations or cracks appearing. After loading to the crack initiation stress, the homogeneity of specimens is destroyed due to differences in particle properties of the heterogeneous structures and stress concentrations occur at the edge of the heterogeneous structures. The maximum principal stress is greater than the bonding limit. Initial cracks in the specimen all occur at the edge of the heterogeneous structures. In this case, the stress is released, the stress concentration degree is reduced, and the stress at the location of crack initiation changes from high to low.
2. As loading continues, more cracks are generated at the edge of the heterogeneous structures. Since the heterogeneous structure is weak, it will reach its bonding limit at a lower load. Therefore, when the damage stress is reached, cracks will only propagate inside the heterogeneous structures and no cracks are generated inside the sandstone matrix. As the loading continuously increases the damage stress, the overall stress in the sandstone specimens increases, cracks grow rapidly inside the heterogeneous structures, and the damage degree increases. The corresponding regional stress of particles was originally in the low-stress region. The moderate and high-stress regions increase due to the overall increasing load. In contrast, for some particles originally in the high-stress region, new cracks begin to occur as the load surpasses the bonding limit, and the corresponding regional stress decreases.
3. After the load reaches the peak strength, overall stress in the sandstone specimen further increases. Cracks continue to develop in the heterogeneous structures with their weaker properties, forming crack groups and cracks also began to occur inside the stronger sandstone matrix. Cracks in the heterogeneous structures and sandstone matrix show a trend of increasing penetration. The high-stress regions, previously relatively dispersed, are now connected, mainly distributed on the left side of the specimen. Since a large number of the generated cracks were mainly concentrated on the right side of the specimen, the stress in this region is released, therefore, the low-stress region is mainly concentrated on the right side.
4. As loading continues, the crack groups propagate and penetrate rapidly, with the main cracks resulting in failure of the specimen. The main cracks penetrate into the sandstone matrix, the matrix morphology is affected by the distribution of the heterogeneous structures, and a large amount of stress is released. Except for a small number of regions on the left side that are still in the high-stress state, most regions transition from a high-stress state to a low-stress state.

At the two characteristic moments of crack initiation and damage, the overall stress of the sandstone specimen with heterogeneous structures is basically the same under different loading rates. Therefore, the number of generated cracks and the crack development and propagation paths are similar and the distribution of maximum principal stress is basically the same. Comparing the two characteristic moments of crack initiation and damage, at the peak stress and final failure, the overall stress of the sandstone specimen with heterogeneous structures increases within different ranges under different loading rates. Furthermore, the greater the loading rate, the greater the stress concentration degree at the edge of the heterogeneous structures, the more completely cracks develop in the heterogeneous structures, and the larger the number of cracks. In this case, the cracks propagate and develop from the upper right region to the lower right region of the specimen, and crack groups form. The crack groups propagate and penetrate rapidly, which leads to failure of the specimen with the formation of main cracks. Finally, the specimen fails and the stress in the whole region is reduced to varying degrees. The stress decreased more significantly on the right side of the specimen (where cracks were generated). The low-stress region, which was originally located in the upper right region of the specimen, gradually moves to the lower right region, and stress in the moderate and high-stress regions on the right side is reduced to some extent. However, a high-stress state is still maintained in a small area on the left side of the specimen.

### Crack evolution time sequence of sandstone specimens with heterogeneous structures

**Figure 7:**
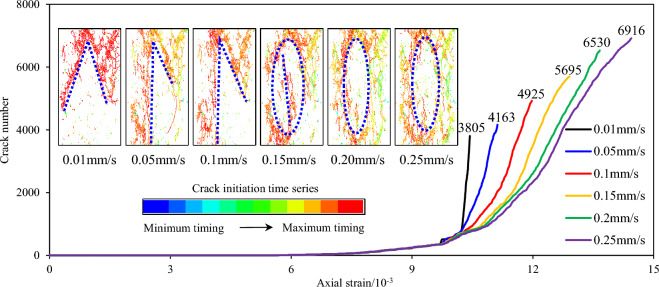
Sequence diagram of crack propagation and final crack number of sandstone specimens with heterogeneous structures under different loading rates.

The timing of the crack generation was monitored using a built-in program in PFC. The timing of crack initiation and propagation was represented by progressive sequences of different colors. The influence of loading rate on the timing of crack evolution in the sandstone specimens was analyzed. Figure 7 shows the variation of the timing of crack evolution and the ultimate total number of cracks in the sandstone specimens with heterogeneous structures under different loading rates.

1. When the loading rate was 0.01 mm/s, it took a relatively long time for the internal structure to adjust to the axial stress due to the relatively low loading rate. Only a few cracks were generated before failure and the rate of crack generation was slow. However, the crack generation rate increased rapidly when the specimen failed, and the final number of cracks was 3805. Due to differences in heterogeneous structures and sandstone matrix particle properties, as the loading rate increases, stress concentration degree at the edge of heterogeneous structures increased. Before failure, more cracks were generated at the edge of the heterogeneous structures than at final failure, the total number of cracks ultimately generated gradually increased, however, the rate of increase slowed down.
2. When the loading rate was 0.01 mm/s, 0.05 mm/s, and 0.1 mm/s, cracks were mainly concentrated in the upper region with a small distribution area. The total number of cracks generated when the specimen failed was small, and the damage degree was relatively low, with the crack group approximately showing an inverted V type. When the loading rate was 0.15 mm/s, the crack group approximately showed a θ type. When the loading rate was 0.20 mm/s and 0.25 mm/s, the total number of cracks due to failure was large, with a high damage degree, and the crack group exhibited an O type. As the loading rate increased, the ultimate failure morphology of the specimen changed from an inverted V type at low loading rates to a θ type at a loading rate of 0.15 mm/s, which gradually evolved into an O type at higher loading rates. The θ type represents the transition from V-type to O-type. When the loading rate was less than 0.15 mm/s, the overall failure degree of the specimen was relatively low; whereas, when the loading rate was higher than 0.15 mm/s, the overall failure degree of the specimen was relatively high.

### Ultimate failure pattern of sandstone specimens with heterogeneous structures

**Figure 8:**
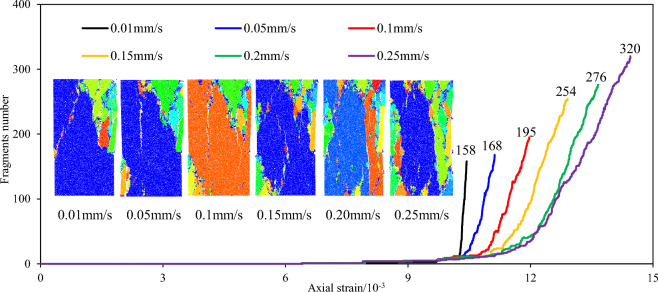
Ultimate failure pattern and number of fractured blocks of sandstone specimens with heterogeneous structures under different loading rates.

Figure 8 shows the variation of the final failure fracture morphology and the number of fragments of the sandstone specimen produced at failure under different loading rates. When the loading rate was 0.01 mm/s, the specimen was broken into 158 pieces due to the relatively low loading rate. The more complete the specimen, the clearer the development path of the main cracks leading to specimen failure and the lower the fragmentation degree. As the loading rate increased, the number of fragments in the final failure stage of the specimen increased from 158 to 320 and the volume of a single fragment gradually decreased. The higher the fragmentation degree, the less clear the development path of the main cracks when the specimen fails. Therefore, the fragmentation degree of the specimen is affected by the loading rate. For the same heterogeneous structures properties and distribution, as the loading rate increases, the stress concentrations at the edge of the heterogeneous structures increase, cracks develop more completely inside the heterogeneous structures, and the fragmentation degree of the specimen at failure is higher.

## Conclusions

1. As the loading rate increased, the peak strength also gradually increased. In addition, the loading rate had a significant influence on the acoustic emission count. As the loading rate increased, the loading lasted for a relatively short period of time, which limited the evolution and propagation of cracks in the heterogeneous structures. As a result, energy continued to accumulate and a large amount of elastic energy was stored in the sandstone. When the peak stress value was reached, the stress was released suddenly, the dissipated energy increased sharply, and the acoustic emission activity became more intense, resulting in instant failure of the sandstone specimen. The larger the loading rate, the greater the dissipated energy and the more energy absorbed by cracks, allowing them to develop and propagate, which leads to a high degree of failure.
2. Under different loading rates, the crack development and propagation and the distribution of maximum principal stress of the sandstone specimens with heterogeneous structures exhibited different behaviors at the moment of crack initiation, damage, peak strength, and final failure. At the characteristic moments of crack initiation and damage, the number of generated cracks, propagation path, and maximum principal stress distribution were not significantly affected by the loading rate. At the peak and final failure moments, more cracks were generated at a higher loading rate. The cracks propagated and developed from the upper right region to the lower right region of the specimen, forming crack groups with similar distribution laws in both the high and low-stress regions. As loading continued, the crack groups rapidly propagated and penetrated the sandstone matrix, leading to the failure of the specimen. Stress in the specimen decreased to varying degrees, especially on the right side of the specimen (where cracks occurred), however, high stress was maintained in a small region on the left side of the specimen.
3. The loading rate had a significant influence on the timing of the crack evolution process and the final failure mode of the sandstone specimens with heterogeneous structures. The time taken by the internal structure to adjust to the axial stress was shortened with the increasing loading rate. Before the final failure, the number of cracks generated at the edge of the heterogeneous structures of the specimen gradually increased, whereas the number of cracks generated at the final failure gradually decreased. The ultimate failure mode of the specimen experienced a transition from an inverted V-type to θ-type, gradually evolving into an O-type failure. The higher the loading rate, the greater the stress concentration at the edge of the heterogeneous structures, the more the ultimate fragments of the specimen, and the greater the damage degree.

## Data Availability

The datasets used and/or analyzed during the current study are available from the corresponding author on reasonable request.

## References

[1] Wang F (2023). Numerical study on sandstone strength and failure characteristics with heterogeneous structure. Bull. Eng. Geol. Environ..

[2] Yin P, Yang S (2018). Spatially varying small-strain stiffness in soils subjected to K–0 loading. Acta Geophys..

[3] Liu G, Cai M, Huang M (2018). Mechanical properties of brittle rock governed by micro–geometric heterogeneity. Comput. Geotech..

[4] Chen S, Ren M, Wang F, Yin D, Chen D (2020). Mechanical properties and failure mechanisms of sandstone with pyrite concretions under uniaxial compression. Geomech. Eng..

[5] Xiao H, He L, Li X, Zhang Q, Li W (2021). Texture synthesis: A novel method for generating digital models with heterogeneous diversity of rock materials and its CGM verification. Comput. Geotech..

[6] Wang F, Jiang B, Chen S, Ren M (2019). Surface collapse control under thick unconsolidated layers by backfilling strip mining in coal mines. Int. J. Rock Mech. Min. Sci..

[7] Wang F, Xu J, Xie J (2019). Effects of arch structure in unconsolidated layers on fracture and failure of overlying strata. Int. J. Rock Mech. Min. Sci..

[8] Chen M (2022). Effects of confining pressure on deformation failure behavior of jointed rock. J. Cent. South Univ..

[9] Qi C, Wang M, Qian Q (2009). Strain–rate effects on the strength and fragmentation size of rocks. Int. J. Impact Eng..

[10] Ye H (2023). Dynamic response characteristics and damage rule of graphite ore rock under different strain rates. Sci. Rep..

[11] Sirdesai NN, Gupta T, Singh TN, Ranjith PG (2018). Studying the acoustic emission response of an Indian monumental sandstone under varying temperatures and strains. Constr. Build. Mater..

[12] Lajtai EZ, Duncan EJS, Carter BJ (1991). The effect of strain rate on rock strength. Rock Mech. Rock. Eng..

[13] Cho SH, Ogata YJ, Kaneko K (2003). Strain–rate dependency of the dynamic tensile strength of rock. Int. J. Rock Mech. Min. Sci..

[14] Wasantha PLP, Ranjith PG, Zhao J, Shao SS, Permata G (2015). Strain rate effect on the mechanical behaviour of sandstones with different grain sizes. Rock Mech. Rock. Eng..

[15] Yin D, Chen S, Xing W, Huang D, Liu X (2018). Experimental study on mechanical behavior of roof–coal pillar structure body under different loading rates. J. China Coal Soc..

[16] Yang H (2021). Investigation of the correlation between crack propagation process and the peak strength for the specimen containing a single pre-existing flaw made of rock–like material. Arch. Civ. Mech. Eng..

[17] Mao R, Mao X, Zhang L, Liu R (2015). Effect of loading rates on the characteristics of thermal damage for mudstone under different temperatures. Int. J. Min. Sci. Technol..

[18] Wen Z, Wang X, Chen L, Lin G, Zhang H (2017). Size effect on acoustic emission characteristics of coal-rock damage evolution. Adv. Mater. Sci. Eng..

[19] Xie S (2020). A damage constitutive model for shear behavior of joints based on determination of the yield point. Int. J. Rock Mech. Min. Sci..

[20] Liu X (2021). Similar simulation study on the deformation and failure of surrounding rock of a large section chamber group under dynamic loading. Int. J. Min. Sci. Technol..

[21] Zhang X (2021). Damage evolution characteristics of saw–tooth joint under shear creep condition. Int. J. Damage Mech..

[22] Ai T, Zhang R, Liu J, Ren L (2012). Space–time evolution rules of acoustic emission location of unloaded coal sample at different loading rates. Int. J. Min. Sci. Technol..

[23] Zhao K (2020). Energy evolution of brittle granite under different loading rates. Int. J. Rock Mech. Min. Sci..

[24] Feng P, Xu Y, Dai F (2021). Effects of dynamic strain rate on the energy dissipation and fragment characteristics of cross–fissured rocks. Int. J. Rock Mech. Min. Sci..

[25] Park CH, Bobet A (2010). Crack initiation propagation and coalescence from frictional flaws in uniaxial compression. Eng. Fract. Mech..

[26] Yu B, Chen Z, Ding Q, Wang L (2016). Non-darcy flow seepage characteristics of saturated broken rocks under compression with lateral constraint. Int. J. Min. Sci. Technol..

[27] Gharbi H, Berest P, Blanco-Martin L, Brouard B (2020). Determining upper and lower bounds for steady state strain rate during a creep test on a salt sample. Int. J. Min. Sci. Technol..

[28] Yin Y, Hu J, Wen G, Xu X, Zeng P (2023). Numerical simulation of micro crack evolution and failure modes of limestone under uniaxial multi-level cyclic loading. Sci. Rep..

[29] Chen S, Yin D, Jiang N, Wang F, Guo W (2019). Simulation study on effects of loading rate on uniaxial compression failure of composite rock-coal layer. Geomech. Eng..

[30] Tang ZC, Wong LNY (2016). Influences of normal loading rate and shear velocity on the shear behavior of artificial rock joints. Rock Mech. Rock Eng..

[31] Zhang X, Zhang Q, Wu S (2017). Acoustic emission characteristics of the rock-like material containing a single flaw under different compressive loading rates. Comput. Geotech..

[32] Huang P, Zhang J, Damascene NJ, Wang ZJ, Li M (2022). Effect of loading rate on mechanical behavior of coal samples with initial damage accumulation. Mech. Time-Depend. Mater..

[33] Zhou Y, Sheng Q, Li N, Fu X (2022). The dynamic mechanical properties of a hard rock under true triaxial damage-controlled dynamic cyclic loading with different loading rates: a case study. Rock Mech. Rock Eng..

[34] Huang Y, Yang S, Zeng W (2016). Experimental and numerical study on loading rate effects of rock–like material specimens containing two unparallel fissures. J. Cent. South Univ..

[35] Shi C, Zhang Q, Wang S (2018). Numerical Simulation Technology and Application with Particle Flow Code (PFC5.0).

[36] Lambert C, Coll C (2014). Discrete modeling of rock joints with a smooth-joint contact model. J. Rock Mech. Geotech. Eng..

[37] Liu S, Ma F, Guo J, Cao J, Wang Z (2020). Numerical study on mesoscopic mechanical behaviors of granite based on multi Pb-GBM method. Chin. J. Rock Mech. Eng..

[38] Potyondy D, Cundall P (2004). A bonded-particle model for rock. Int. J. Rock Mech. Min. Sci..

